# Tandem Multimerization Can Enhance the Structural Homogeneity and Antifungal Activity of the Silkworm Protease Inhibitor BmSPI39

**DOI:** 10.3390/cells12050693

**Published:** 2023-02-22

**Authors:** Youshan Li, Yuan Wang, Rui Zhu, Xi Yang, Meng Wei, Zhaofeng Zhang, Changqing Chen, Ping Zhao

**Affiliations:** 1 College of Biological Science and Engineering, Shaanxi University of Technology, Hanzhong 723001, Shaanxi Province, China; 2 Qinba Mountain Area Collaborative Innovation Center of Bioresources Comprehensive Development, Hanzhong 723001, Shaanxi Province, China; 3 Qinba State Key Laboratory of Biological Resources and Ecological Environment (Incubation), Shaanxi University of Technology, Hanzhong 723001, Shaanxi Province, China; 4 Shaanxi Province Key Laboratory of Bio-Resources, Hanzhong 723001, Shaanxi Province, China; 5 State Key Laboratory of Silkworm Genome Biology, Southwest University, Chongqing 400715, China

**Keywords:** protease inhibitor, tandem multimer, antifungal activity, pathogenic fungi, conidial germination, *Bombyx mori*

## Abstract

Previous studies have shown that BmSPI39, a serine protease inhibitor of silkworm, can inhibit virulence-related proteases and the conidial germination of insect pathogenic fungi, thereby enhancing the antifungal capacity of *Bombyx mori*. The recombinant BmSPI39 expressed in *Escherichia coli* has poor structural homogeneity and is prone to spontaneous multimerization, which greatly limits its development and application. To date, the effect of multimerization on the inhibitory activity and antifungal ability of BmSPI39 remains unknown. It is urgent to explore whether a BmSPI39 tandem multimer with better structural homogeneity, higher activity and a stronger antifungal ability can be obtained by protein engineering. In this study, the expression vectors of BmSPI39 homotype tandem multimers were constructed using the isocaudomer method, and the recombinant proteins of tandem multimers were obtained by prokaryotic expression. The effects of BmSPI39 multimerization on its inhibitory activity and antifungal ability were investigated by protease inhibition and fungal growth inhibition experiments. In-gel activity staining and protease inhibition assays showed that tandem multimerization could not only greatly improve the structural homogeneity of the BmSPI39 protein, but also significantly increase its inhibitory activity against subtilisin and proteinase K. The results of conidial germination assays showed that tandem multimerization could effectively enhance the inhibitory ability of BmSPI39 on the conidial germination of *Beauveria bassiana*. A fungal growth inhibition assay showed that BmSPI39 tandem multimers had certain inhibitory effects on both *Saccharomyces cerevisiae* and *Candida albicans*. The inhibitory ability of BmSPI39 against these the above two fungi could be enhanced by tandem multimerization. In conclusion, this study successfully achieved the soluble expression of tandem multimers of the silkworm protease inhibitor BmSPI39 in *E. coli* and confirmed that tandem multimerization can improve the structural homogeneity and antifungal ability of BmSPI39. This study will not only help to deepen our understanding of the action mechanism of BmSPI39, but also provide an important theoretical basis and new strategy for cultivating antifungal transgenic silkworms. It will also promote its exogenous production and development and application in the medical field.

## 1. Introduction

*Bombyx mori* is a silk-spinning insect with great economic value that has accumulated a large amount of basic research and has become one of the best models of insect biochemistry, genetics and genomics [1,2,3,4]. After thousands of years of artificial domestication, although the silkworm has acquired some characteristics conducive to production, such as being suitable for social rearing and developing neatly, it has also accumulated unfavorable characteristics that are vulnerable to infection by pathogenic microorganisms. Insect pathogenic fungi, as a new type of biological pesticide, have been widely used in agriculture and forestry pest control and mosquito control. The use of fungal biopesticides will inevitably cause cross-infection with silkworms, which will lead to highly pathogenic silkworm disease, seriously affect the yield and quality of cocoon silk and cause significant economic losses to the whole sericulture industry. Therefore, it is of great significance for the whole silkworm production to elucidate the defense mechanism of silkworms against fungal diseases, seek new defense measures and innovate silkworm genetic materials.

Entomopathogenic fungi penetrate the insect integument through the combined action of mechanical pressure and enzyme degradation [5,6,7]. Many entomopathogenic fungi can secrete subtilisin-like proteases, an important class of cuticle-degrading proteases. Cuticle-degrading proteases are important virulence factors, which are usually secreted in insect integument during spore germination and participate in the penetration process of host cuticles [8,9]. The overexpression of these toxic protease can significantly enhance the virulence of pathogenic fungi [10,11,12]. Unlike mammals, insects lack lymphocytes or immunoglobulins, and serine protease inhibitors are considered to play an important role in insect immunity [13,14,15]. Our previous studies systematically identified immune-related silkworm protease inhibitors and found that many protease inhibitors with the trypsin inhibitor-like cysteine-rich domain (TIL) were upregulated after microbial infection, implying that TIL-type protease inhibitors may be involved in the immune process of silkworms [16,17]. Two structurally unique TIL-type protease inhibitors, BmSPI38 and BmSPI39, can block the harmful melanization induced by cuticle-degrading protease 1 (CDEP-1) and inhibit the conidial germination of *Beauveria bassiana*, thereby enhancing the antifungal ability of silkworms [18,19]. In addition, studies have found that many protease inhibitors, especially TIL protease inhibitors, can be secreted into the cocoon layer during silk secretion and provide effective protection for the cocoon shell and pupa by inhibiting the activities of exogenous proteases secreted by pathogenic microorganisms [20,21]. These results indicate that such inhibitors can be used as fungal resistance factors in the field of medicine and agriculture.

Our previous study found that the recombinant protease inhibitor BmSPI39 is prone to spontaneous polymerization, forming dimers, trimers and tetramers [22,23]. Further research has shown that BmSPI39 mainly exists and functions in the form of a tetramer in silkworm tissues, rather than a monomer [23]. To date, the activity and function of BmSPI39 are well understood, but the effect of multimerization on its inhibitory activity and antifungal ability remains unknown. In addition, the structural homogeneity of recombinant protein expressed by the single-copy BmSPI39 gene is poor, which greatly limits its development and application. It is urgent to explore whether BmSPI39 tandem multimeric protein with better structural homogeneity, higher activity and a stronger antifungal ability can be obtained by protein engineering.

In this study, we intend to construct the expression vectors of BmSPI39 homomeric tandem multimers; obtain the recombinant multimeric protein using the prokaryotic expression system; screen the tandem multimeric proteins with better structural homogeneity, stronger activity and higher exogenous expression level; and explore the influence of multimerization on its inhibitory activity and antifungal ability. This study will not only help to deepen the understanding of the action mechanism of BmSPI39 and provide an important theoretical basis and new strategies for cultivating antifungal transgenic silkworms, but it will also promote its exogenous production and development and application in the medical field.

## 2. Materials and Methods

### 2.1. Fungi and Reagents

TransStart^®^ TopTaq DNA Polymerase was purchased from TransGen Biotech (Beijing, China). *Nde* I, *Not* I, *Bam*H I and *Bgl* II endonuclease were purchased from Takara (Dalian, China). Proteinase K from *Tritirachium album* limber were purchased from Roche (Mannheim, Germany). Subtilisin A from *Bacillus licheniformis*, N-acetyl-D,L-phenylalanine-β-naphthylester and Fast Blue B Salt were purchased from Sigma-Aldrich (St. Louis, MO, USA). Fluorescein isothiocyanate (FITC)-labeled casein was purchased from Thermo Fisher Scientific (Waltham, MA, USA). *Escherichia coli* BL21(DE3) competent cells were purchased from Sangon Biotech (Shanghai, China). *E. coli* Origami 2(DE3), *B. bassiana*, *Saccharomyces cerevisiae* and *Candida albicans* were all preserved by the College of Biological Science and Engineering, Shaanxi University of Technology.

### 2.2. Vector Construction of the Basic Units

A PCR amplification was performed using a previously constructed *BmSPI3*9-p28 plasmid as a template, *BmSPI39*-*Nde* I-*Bam*H I-F as the upstream primer and *BmSPI39*-*Not* I-R, *BmSPI39*-*Bgl* II-R or *BmSPI39*-*L*-*Bgl* II-R as the downstream primers (Table 1). The conditions of the PCR were as follows: predenaturation at 94 °C for 3 min; 30 cycles of denaturation at 94 °C for 30 s, annealing at 58 °C for 30 s, extension at 72 °C for 30 s; final extension at 72 °C for 10 min. The PCR products were detected by electrophoresis using 1.5% agarose gel. The target gene fragments *Nde* I/*Bam*H I-*SPI39*-*Not* I (255 bp), *Nde* I/*Bam*H I-*SPI39*-*Bgl* II (248 bp) and *Nde* I/*Bam*H I-*SPI39L*-*Bgl* II (293 bp) were recovered and ligated into the pEASY-T1 simple cloning vector (TransGen Biotech) and then transformed into *E. coli* Trans-T1 component cells (TransGen Biotech). The positive clones obtained by colony PCR screening were verified by sequencing.

### 2.3. Expression Vector Construct of BmSPI39 Tandem Multimers

The *Nde* I/*Bam*H I-*SPI39*-*Not* I plasmid and p28 expression vector were double digested by *Nde* I and *Not* I, and then the *Nde* I/*Bam*H I-*SPI39*-*Not* I-p28 plasmid was constructed under the action of the T4 ligase (Takara). The recombinant vector was named the His_6_-*SPI39*-monomer expression vector. *Bam*H I (G/GATCC) and *Bgl* II (A/GATCT) are a pair of isocaudarners. The basic unit vectors *Nde* I/*Bam*H I-*SPI39*-*Bgl* II and *Nde* I/*Bam*H I-*SPI39L*-*Bgl* I were double digested with *Nde* I and *Bgl* I, while the constructed His_6_-*SPI39*-monomer expression vector was double digested with *Nde* I and *Bam*H I. Two tandem dimer expression vectors, His_6_-*SPI39*-dimer and His_6_-*SPI39L*-dimer, were obtained by ligating the recovered *Nde* I/*Bam*H I-*SPI39*-*Bgl* II and *Nde* I/*Bam*H I-*SPI39L*-*Bgl* II fragments with His_6_-*SPI39*-monomer vector fragments, respectively. Similarly, the homo-trimer expression plasmids (His_6_-*SPI39*-trimer and His_6_-*SPI39L*-trimer) can be constructed by ligating *Nde* I/*Bam*H I-*SPI39*-*Bgl* II and *Nde* I/*Bam*H I-*SPI39L*-*Bgl* II fragments into the dimer expression plasmids (His_6_-*SPI39*-dimer and His_6_-*SPI39L*-dimer) using the isocaudomer method, respectively. The homo-tetramer expression plasmids (His_6_-*SPI39*-tetramer and His_6_-*SPI39L*-tetramer) were constructed on the basis of homo-trimer expression plasmids (His_6_-*SPI39*-trimer and His_6_-*SPI39L*-trimer). The constructed expression plasmid of BmSPI39 homo-tandem multimers were verified by double enzyme digestion using endonuclease *Nde* I and *Not* I and were sent to the company for sequencing verification.

### 2.4. Protein Expression and Purification

The constructed expression plasmids of the BmSPI39 tandem multimers were transformed into *E. coli* BL21(DE3) and Origami 2(DE3) strains and expressed as fusion proteins with 12 residues of a polyhistidine tag (MGHHHHHHMGGS) at the N-terminus. When the OD_600_ of the culture reached 0.6–1.0, the protein expression was induced with 0.2 mmol/L IPTG at 37 °C for 5 h or at 16 °C for 20 h. After centrifugation at 6000 rpm for 30 min, the *E. coli* cells were collected and suspended with a binding buffer (20 mmol/L Tris-HCl, 500 mmol/L NaCl, pH 7.9). After ultrasonic crushing and centrifugation, the supernatant of the bacteria was collected and analyzed using 16.5% SDS-PAGE. The recombinant proteins from the BL21(DE3) cells were purified with Ni^2+^-NTA (nitrilotriacetic acid) affinity chromatography (Sangon Biotech, Shanghai, China). The supernatant containing recombinant protein was filtered with a 0.45 μm filter membrane and then loaded onto a 1 mL Ni^2+^-NTA affinity chromatography column. The flow rate was controlled at 0.5–1.0 mL/min. The column was washed and eluted sequentially by a binding buffer supplemented with 0, 20, 50, 100 and 400 mmol/L imidazole until no more protein was eluted. The eluted portion of the highly enriched target protein was collected based on 16.5% SDS-PAGE. The second round of immobilized-nickel affinity chromatography was performed after the removal of imidazole by dialysis. After electrophoretic detection, the pure proteins of tandem multimers were finally collected and dialyzed to a 20 mmol/L PBS buffer (pH 7.8) for storage.

### 2.5. In-Gel Activity Staining of Protease Inhibitor

The induced expression protein samples were mixed with a 4× Native PAGE loading buffer (40 mmol/L Tris-HCl pH 8.0, 40% glycerol, 0.032% bromophenol blue) and separated by 10% Native PAGE, followed by the in-gel activity staining of the protease inhibitor. The in-gel activity staining of the protease inhibitors referred to the previous reported method [24]. The electrophoresed gel was placed in a protease solution and was incubated for 30 min at 37 °C, 45 rpm, without light. After recovering the protease solution, the gel was washed with ddH_2_O and then left for 30 min at 37 °C in dark conditions. At a volume ratio of 1:10, the mixture of the substrates solution (200 mg N-acetyl-D,L-phenylalanine-β-naphthylester dissolved in 100 mL of N,N′-dimethylformamide) and staining solution (100 mg of Fast Blue B Salt dissolved in 100 mL of 0.1 mol/L pH 8.0 Tris-HCl buffer containing 20 mmol/L CaCl_2_) were added and incubated for 15 min at 37 °C, 45 rpm. Subsequently, the staining solution was discarded, and the gel was washed with ddH_2_O to terminate the reaction. The gels were stained fuchsia due to the diazotization coupling reaction of β-naphthol that was produced by protease hydrolyzed N-acetyl-D,L-phenylalanine-β-naphthylester on the gel [25,26]. If the protease inhibitor in the gel can inhibit the corresponding protease activity, its location will not be stained and will appear as a white band.

### 2.6. Protease Inhibition Assays

The moles of the BmSPI39 tandem multimeric proteins were converted to the moles of TIL domains based on the number of TIL domains in the protease inhibitor molecules. For example, 1 mol His_6_-SPI39-dimer and His_6_-SPI39L-dimer proteins have 2 mol TIL domains. A total of 0.003 nmol of subtilisin (MW 27 kDa) or proteinase K (MW 28.8 kDa) was mixed with the protease inhibitor and supplemented with a buffer (100 mmol/L Tris-HCl, 20 mmol/L CaCl_2_, pH 8.0) to a final volume of 100 μL. Then, it was incubated at 37 °C for 30 min. The molar ratio of the TIL domain of the inhibitor to protease was set as 0.5, 1, 2, 5, 10 and 15. Then, 100 μL of 10 μg/mL FITC-casein was added and incubated at 37 °C for 60 min in the dark. The fluorescence intensity was measured at a 485 nm excitation and 528 nm emission wavelength, and the residual enzyme activity was calculated. The inhibitory activity of the protease inhibitor against protease was assessed with the following formula: residual enzyme activity% = enzyme activity of experimental group/enzyme activity of control group × 100%.

### 2.7. Assays of Conidial Germination of B. bassiana

*B. bassiana* was inoculated in a solid potato dextrose agar (PDA) medium and cultured for 10 days at 28 °C. Spores were collected and mycelium was removed by filtration with sterile absorbent cotton. A conidial suspension was prepared at a concentration of 9 × 10^7^ conidia/mL using sterilized ddH_2_O. A total of 200 μL of a potato dextrose liquid (PDL) medium was mixed with 100 μL of a 0.03 nmol/µL TIL domain equivalent protease inhibitor, and then 100 μL of a conidial suspension was added and incubated at 28 °C and 80 rpm for 4 h, 8 h and 12 h. The control group was treated with an equal volume of 20 mmol/L PBS. The conidial germination rate was calculated via microscopic observations after different incubation times. A conidium was considered germinated when the length of its germination tube was greater than or equal to its width. All the experiments were repeated three times. The conidial germination rate% = number of germinated conidia/total number of conidia × 100%.

### 2.8. Fungal Growth Inhibition Assay

*C. albicans* or *S. cerevisiae* cultured overnight were inoculated into the potato dextrose liquid medium at a ratio of 1:1000 and were cultured at 28 °C and 100 rpm for 24 h. Fungal cultures were filtered using sterile absorbent cotton and then centrifuged at 4 °C and 4000 g for 20 min to collect the spores. After cell counting, 1 × 10^5^ spores/mL spore suspensions were prepared using sterile ddH_2_O. In total, a 160 µL spore suspension, 160 µL PDL medium and 160 µL of a 0.01 nmol/µL TIL domain equivalent protease inhibitor were mixed in a 2.0 mL centrifuge tube. A 20 mmol/L PBS was used as the negative control, and 100 mmol/L EDTA was used as a positive control. The mixture was incubated by shaking at 28 °C and 100 rpm. After incubation for 0 h, 12 h, 24 h, 36 h and 48 h, 100 µL of the culture was taken into the 96-well plate. The absorbance at 600 nm was measured, and the fungal growth kinetics curve was drawn. All the assays were repeated three times. The fungal growth inhibition rate was calculated according to the following formula: Inhibitory rate% = (1 − OD_600exp_/OD_600pbs_) × 100%.

### 2.9. Statistical Analysis

All statistical analyses of the data were performed using the Data Processing System (DPS) software version 9.01. Statistically significant differences were assessed by using a one-way analysis of variance (ANOVA). The error bar represents the standard error of the mean (n = 3). Different lowercase letters “a to e” indicate significant differences between the treatment groups at *p* < 0.05, while those marked with one identical letter mean no significant differences between the treatment groups at *p* < 0.05.

## 3. Results

### 3.1. Design and Construction of Expression Vector of BmSPI39 Tandem Multimers

**Figure 1:**
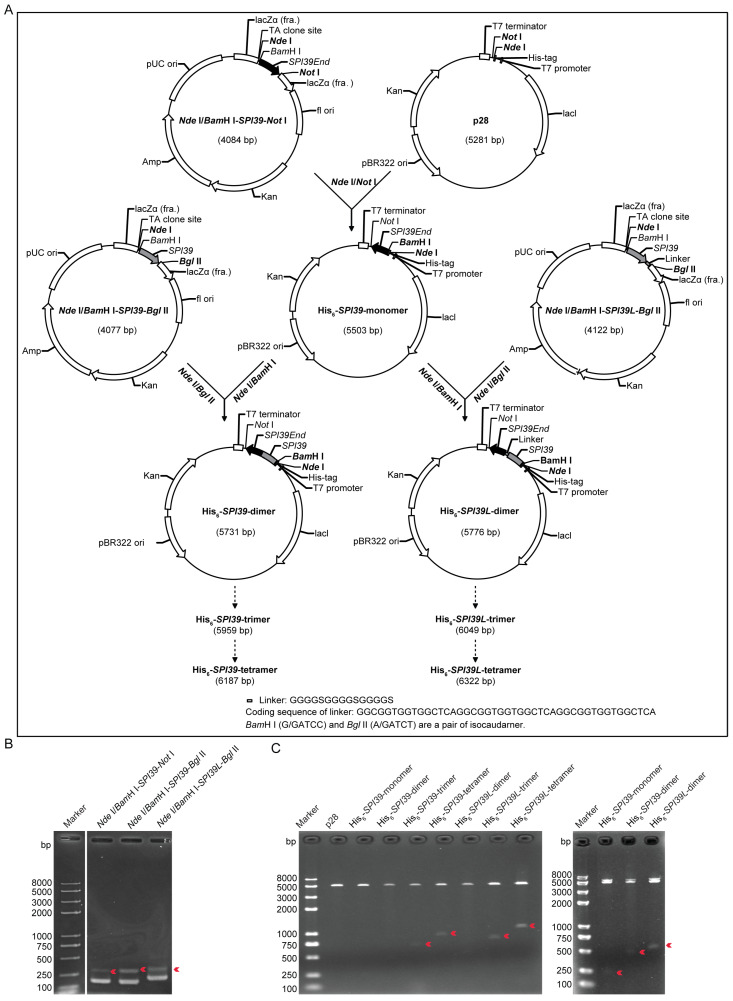
Design and construction of expression vector of BmSPI39 tandem multimers. (**A**) Schematic diagram of expression vector construction of BmSPI39 tandem multimers. Glycine-rich flexible linker was used to connect protein modules. “*SPI39*” represents the coding sequence of BmSPI39 protein module, and “*SPI39L*” represents the coding sequence of BmSPI39 protein module connected by flexible linker. The amino acid sequence of linker is “GGGGSGGGGSGGGGS”. The coding sequence of linker is “GGCGGTGGTGGCTCAGGCGGTGGTGGCTCAGGCGGTGGTGGCTCA”. *Bam*H I (G/GATCC) and *Bgl* II (A/GATCT) are a pair of isocaudarners. (**B**) Agarose gel electrophoresis detection of PCR products of basic unit fragments. Target products of the PCR are indicated by red arrows. (**C**) Double digestion of recombinant expression vector using *Nde* I/*Not* Ienzymes. p28 is a derivative expression plasmid of pET28b. The target fragments produced by double digestion are shown by red arrows. The bands of about 5000 bp are linearized vector fragments produced by double digestion.

In order to obtain the active proteins of the BmSPI39 homologous tandem multimers, two sets of expression vector construction schemes were designed (Figure 1A). In the first strategy, a flexible linker sequence was added between the fusion proteins. The second strategy did not add a linker sequence between the fusion proteins. A linker is an amino acid chain that connects two fusion proteins. It has a certain flexibility that helps the protein fold correctly during protein expression, allowing the proteins on both sides to perform their independent functions [27,28]. Glycine-rich flexible linkers such as (GGGGS)n are the most commonly used linkers for separating different parts of the fusion protein. The linker sequence used here was “GGGGSGGGGSGGGGS”, and its corresponding coding sequence was “GGCGGTGGTGGCTCAGGCGGTGGTGGCTCAGGCGGTGGTGGCTCA”. Firstly, the basic unit vector *Nde* I/*Bam*H I-*SPI39*-*Not* I, *Nde* I/*BamH* I-*SPI39*-*Bgl* II and *Nde* I/*Bam*H I-*SPI39L*-*Bgl* II were constructed. Then, the gene fragment “*Nde* I/*Bam*H I-*SPI39*-*Not* I” was inserted into a p28 expression vector by double-enzyme digestion to construct the His_6_-*SPI39*-monomer expression vector. Next, the gene fragments *Nde* I/*Bam*H I-*SPI39*-*Bgl* II and *Nde* I/*Bam*H I-*SPI39L*-*Bgl* II were inserted into the *Nde* I/*Bam*H I sites of the plasmid His_6_-*SPI39*-monomer by using the isocaudomer method, respectively. The recombinant plasmids His_6_-*SPI39*-dimer and His_6_-*SPI39L*-dimer were obtained. Finally, homo-trimer expression plasmids (His_6_-*SPI39*-trimer and His_6_-*SPI39L*-trimer) and homo-tetramer expression plasmids (His_6_-*SPI39*-tetramer and His_6_-*SPI39L*-tetramer) were constructed by using the isocaudomer method.

To obtain gene fragments of *Nde* I/*Bam*H I-*SPI39*-*Not* I (255 bp), *Nde* I/*Bam*H I-*SPI39*-*Bgl* II (248 bp) and *Nde* I/*Bam*H I-*SPI39L*-*Bgl* II (293 bp), a PCR amplification was performed using *BmSPI39*-*Nde* I-*Bam*H I-F as the upstream primer and *BmSPI39*-*Not* I-R, *BmSPI39*-*Bgl* II-R or *BmSPI39*-*L*-*Bgl* II-R as the downstream primers. The PCR products were detected by using agarose gel electrophoresis. Three specific target bands were detected at their expected sizes on agarose gels (Figure 1B). The above three gene fragments were cloned into a pEASY-T1 simple cloning plasmid, and the positive clones were screened by colony PCR. The expression vectors of BmSPI39 tandem multimers were constructed as shown in Figure 1A. The results of *Nde* I/*Not* I double digestion and sequencing showed that the expression vectors His_6_-*SPI39*-monomer, His_6_-*SPI39*-dimer, His_6_-*SPI39*-trimer, His_6_-*SPI39*-tetramer, His_6_-*SPI39L*-dimer, His_6_-*SPI39L*-trimer and His_6_-*SPI39L*-tetramer were successfully constructed (Figure 1C).

### 3.2. Protein Expression and Purification of BmSPI39 Tandem Multimers

**Figure 2:**
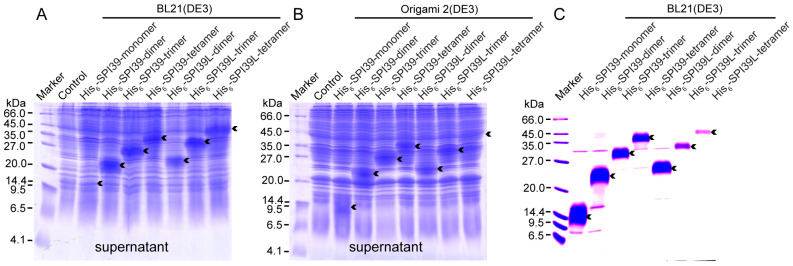
Protein expression and purification of BmSPI39 tandem multimers. (**A**) SDS-PAGE analysis of BmSPI39 tandem multimers expressed in BL21(DE3) cells; (**B**) SDS-PAGE analysis of BmSPI39 tandem multimers expressed in Origami 2(DE3) cells; (**C**) SDS-PAGE analysis of the purified BmSPI39 tandem multimers. The *E. coli* cells transformed with p28 plasmid were used as the control. “Supernatant” indicates the supernatant part of *E. coli* cells. Arrows represent the fusion proteins of BmSPI39 tandem multimers. In addition to the target protein bands indicated by the arrows, some weak bands were found, which may be the impurity proteins coeluted with the target protein.

In order to obtain sufficient BmSPI39 tandem multimeric proteins for subsequent research, the constructed expression vectors were transformed into *E. coli* cells for induced expression. The protein samples extracted from BL21(DE3) and Origami 2 cells were separated using a 16.5% SDS-PAGE (Figure 2A,B). The theoretical molecular weights of His_6_-SPI39-monomer, His_6_-SPI39-dimer, His_6_-SPI39-trimer and His_6_-SPI39-tetramer are 9506.58, 17894.94, 26283.30 and 34671.67 Da, respectively. The theoretical molecular weights of His_6_-SPI39L-dimer, His_6_-SPI39L-trimer and His_6_-SPI39L-tetramer are 18840.80, 28175.02 and 37509.24 Da, respectively. The SDS-PAGE results showed that the BmSPI39 tandem multimeric proteins were expressed in the soluble form in both the BL21(DE3) and Origami 2(DE3) strains, and the band position of the target protein showed a step-like change with increasing molecular weight. His_6_-SPI39-dimer, His_6_-SPI39-trimer, His_6_-SPI39-tetramer, His_6_-SPI39L-dimer, His_6_-SPI39L-trimer and His_6_-SPI39L-tetramer were highly expressed in the supernatants of BL21(DE3) and Origami 2(DE3). The expression of His_6_-SPI39-monomer was low in the supernatant of BL21(DE3) and relatively high in the supernatant of Origami 2(DE3). Overall, BL21(DE3) was more suitable for expressing BmSPI39 tandem multimeric proteins than Origami 2(DE3).

In order to obtain the pure protein of the BmSPI39 tandem multimeric proteins, BL21(DE3) harboring recombinant expression vectors were mass cultured and induced for expression. The target proteins were purified by two repeats of Ni^2+^-NTA affinity chromatography. The SDS-PAGE results showed that the obtained His_6_-SPI39-monomer, His_6_-SPI39-dimer, His_6_-SPI39-trimer, His_6_-SPI39-tetramer, His_6_-SPI39L-dimer, His_6_-SPI39L-trimer and His_6_-SPI39L-tetramer proteins had high purity and could meet the requirements of subsequent experiments (Figure 2C).

### 3.3. Activity and Structural Homogeneity Analysis of BmSPI39 Tandem Multimers

**Figure 3:**
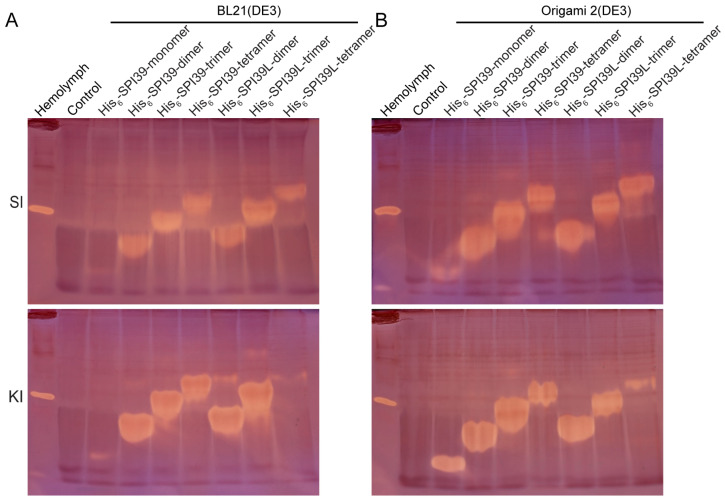
Activity and structural homogeneity analysis of BmSPI39 tandem multimers using in-gel activity staining. (**A**) Activity analysis of BmSPI39 tandem multimers expressed in BL21(DE3) cells; (**B**) activity analysis of BmSPI39 tandem multimers expressed in Origami 2(DE3) cells. “SI” and “KI” indicate subtilisin inhibitors and proteinase K inhibitors, respectively. The supernatant part of *E. coli* cells transformed with p28 plasmid was used as a negative control. The hemolymph of the fifth instar larvae of the silkworm contains a variety of protease inhibitors, so it can be used as a positive control to detect whether the in-gel activity staining of protease inhibitor is successful.

To explore the effect of tandem multimerization on the activity and structural homogeneity of BmSPI39, the tandem BmSPI39 multimers expressed in BL21(DE3) and Origami 2(DE3) were analyzed by in-gel activity staining (Figure 3A,B). The results of the activity staining showed that all BmSPI39 tandem multimeric proteins expressed in the two strains had inhibitory activity against subtilisin and proteinase K. As the number of tandem units increased, the inhibitory bands of the recombinant proteins showed a stepped distribution. A strong active band was mainly detected for each tandem protein, while an extremely weak active band was also detected above the main active bands of His_6_-SPI39-dimer, His_6_-SPI39-trimer, His_6_-SPI39-tetramer, His_6_-SPI39L-dimer, His_6_-SPI39L-trimer and His_6_-SPI39L-tetramer. Under the same treatment and loading conditions, His_6_-SPI39-dimer, His_6_-SPI39-trimer, His_6_-SPI39-tetramer, His_6_-SPI39L-dimer, His_6_-SPI39L-trimer and His_6_-SPI39L-tetramer expressed in *E. coli* cells showed far stronger inhibitory activities against subtilisin than His_6_-SPI39-monomer. The tandem multimeric proteins, except for His_6_-SPI39L-tetramer, also exhibited more potent inhibitory activities against proteinase K than His_6_-SPI39-monomer. His_6_-SPI39-monomer showed a stronger inhibitory activity on proteinase K than it did on subtilisin, while His_6_-SPI39L-tetramer showed a stronger inhibitory activity towards subtilisin than it did towards proteinase K (Figure 3B). In general, the inhibitory activities of the tandem multimeric proteins expressed in Origami 2(DE3) were stronger than that expressed in BL21(DE3), but the active forms expressed in BL21(DE3) were more uniform. The above results indicate that tandem multimerization based on protein engineering can greatly improve the inhibitory activity and structural homogeneity of BmSPI39.

### 3.4. Comparison of Inhibitory Capacity of BmSPI39 Tandem Multimers against Microbial Protease

**Figure 4:**
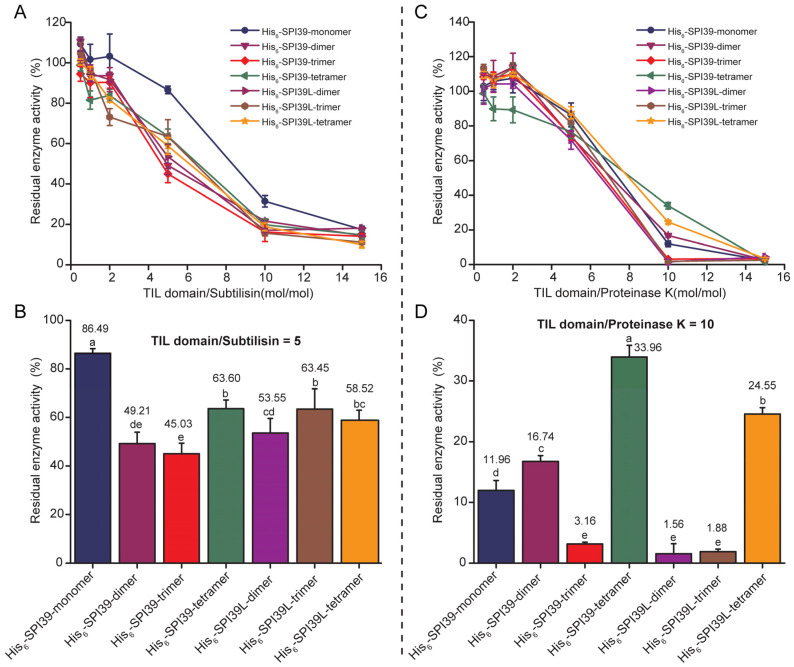
Comparison of inhibitory capacity of BmSPI39 tandem multimers against microbial protease using protease inhibition assays. (**A**) Inhibitory effects of increasing concentrations of BmSPI39 tandem multimers against subtilisin A from *B. licheniformis*; (**B**) inhibitory activities of BmSPI39 tandem multimers against subtilisin when the ratio of TIL domain to protease is 5; (**C**) inhibitory effects of increasing concentrations of BmSPI39 tandem multimers against proteinase K from *E. album*; (**D**) inhibitory activities of BmSPI39 tandem multimers against proteinase K when the ratio of TIL domain to protease is 10. Error bars represent the standard error of the mean (*n* = 3). Different letters “a–e” indicate a significant difference between groups (*p* < 0.05), and one identical letter indicates no significant difference between groups (*p* < 0.05).

To further explore the effect of tandem multimerization on BmSPI39 activity, subtilisin and proteinase K were selected for protease inhibition activity assays. The residual enzyme activities of the proteases treated by seven forms of protease inhibitors were determined under the same molar equivalent of the TIL domain. The results showed that the inhibitory activities of His_6_-SPI39-dimer, His_6_-SPI39-trimer, His_6_-SPI39-tetramer, His_6_-SPI39L-dimer, His_6_-SPI39L-trimer and His_6_-SPI39L-tetramer towards subtilisin were significantly stronger than His_6_-SPI39-monomer (Figure 4A,B). Among them, His_6_-SPI39-trimer had the strongest inhibitory activity against subtilisin (Figure 4B). His_6_-SPI39-monomer showed stronger inhibition towards proteinase K than His_6_-SPI39-dimer, His_6_-SPI39-tetramer and His_6_-SPI39L-tetramer, but it was significantly weaker than His_6_-SPI39-trimer, His_6_-SPI39L-dimer and His_6_-SPI39L-trimer (Figure 4C,D). There was no significant difference in the inhibitory activity towards proteinase K between His_6_-SPI39-trimer, His_6_-SPI39L-dimer and His_6_-SPI39L-trimer (Figure 4C,D). Overall, His_6_-SPI39-trimer showed the strongest inhibitory activity against subtilisin and proteinase K. The above results indicate that tandem multimerization can greatly improve the inhibitory activity of BmSPI39 against proteases, and each tandem protein has a certain preference for inhibiting different proteases.

### 3.5. Evaluation of Inhibitory Ability of BmSPI39 Tandem Multimers on Conidial Germination of Silkworm Pathogenic Fungi B. bassiana

**Figure 5:**
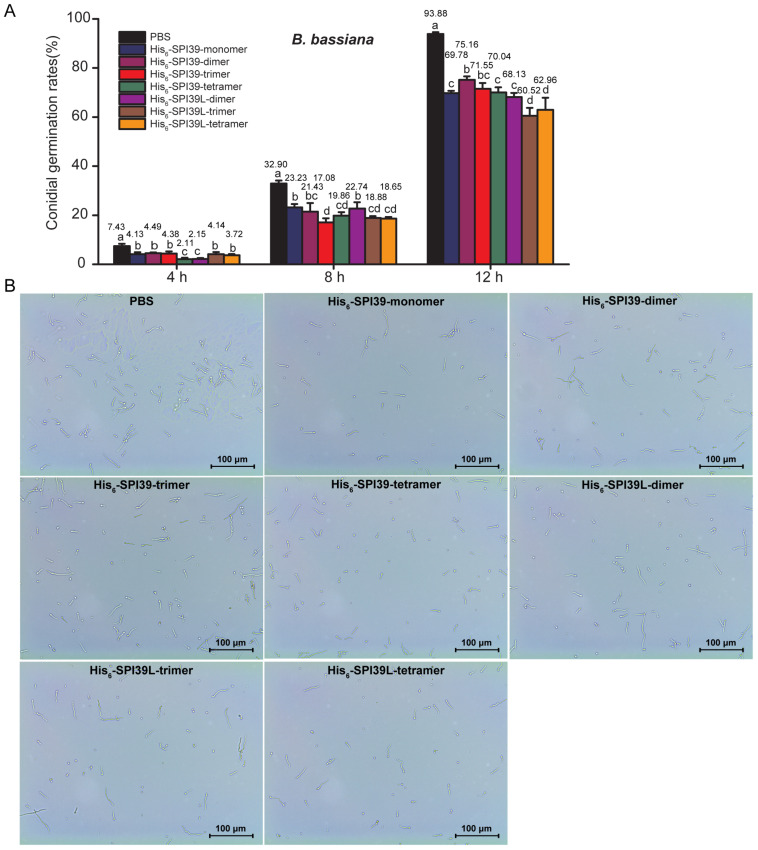
Evaluation of inhibitory ability of protease inhibitors on *B. bassiana*. (**A**) Inhibitory effects of BmSPI39 tandem multimers on conidial germination; (**B**) microscopic observation of conidial germination in different treatment groups after incubation for 12 h. The control group was treated with equal volume of 20 mmol/L PBS. Error bars represent the standard error of the mean (*n* = 3). Different letters “a–d” indicate a significant difference between groups (*p* < 0.05), and one identical letter indicates no significant difference between groups (*p* < 0.05).

To evaluate the inhibitory ability of BmSPI39 tandem multimeric proteins on the conidial germination of *B. bassiana*, an important pathogenic fungus of *B. mori*, conidia, was incubated with protease inhibitors containing the same molar equivalent of the TIL domain. The results showed that all forms of tandem proteins could significantly inhibit the conidial germination of *B. bassiana* (Figure 5A,B). His_6_-SPI39-trimer, His_6_-SPI39-tetramer, His_6_-SPI39L-trimer and His_6_-SPI39L-tetramer inhibited conidial germination more effectively than His_6_-SPI39-monomer after incubation for 8 h. After incubation for 12 h, the conidial germination rate of the PBS treatment group had reached 93.88%, while that of the His_6_-SPI39-monomer, His_6_-SPI39-dimer, His_6_-SPI39-trimer, His_6_-SPI39-tetramer, His_6_-SPI39L-dimer, His_6_-SPI39L-trimer and His_6_-SPI39L-tetramer treatment groups were 69.78%, 75.16%, 71.55%, 70.04%, 68.13%, 60.52% and 62.96%, respectively. His_6_-SPI39L-trimer and His_6_-SPI39L-tetramer still showed stronger inhibition on conidial germination than His_6_-SPI39-monomer, while His_6_-SPI39-dimer showed less inhibition on conidial germination than His_6_-SPI39-monomer. There was no significant difference between the His_6_-SPI39-trimer, His_6_-SPI39-tetramer, His_6_-SPI39L-dimer and His_6_-SPI39-monomer treatment groups. The above results indicate that tandem multimerization can effectively enhance the ability of BmSPI39 to inhibit the conidial germination of *B. bassiana*.

### 3.6. Evaluation of the Inhibitory Effects of BmSPI39 Tandem Multimers on the Growth of Single-Celled Fungus Saccharomyces cerevisiae and Opportunistic Human Pathogen Candida albicans

**Figure 6:**
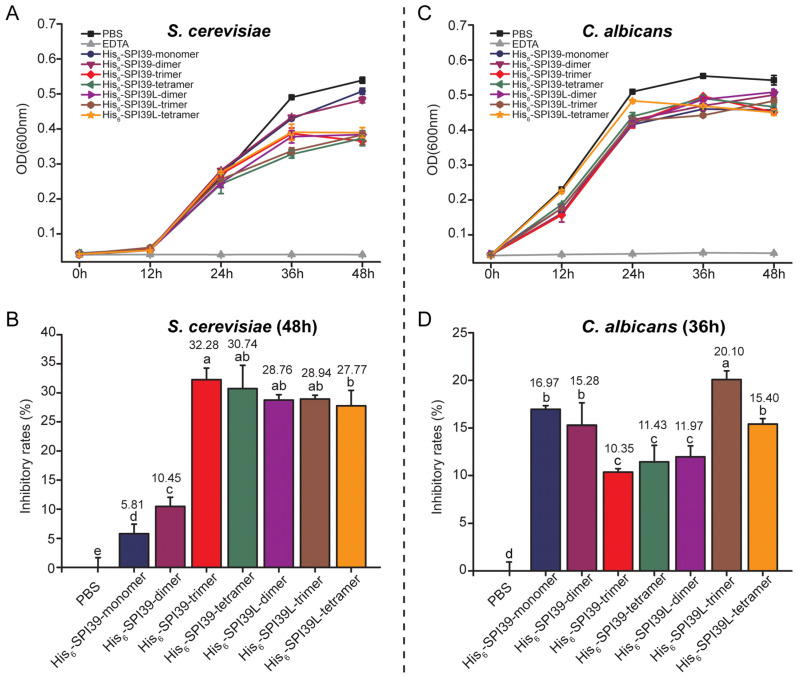
Evaluation of the inhibitory effects of protease inhibitors on single-celled fungus *S. cerevisiae* and opportunistic human pathogen *C. albicans*. (**A**) Inhibitory effects of BmSPI39 tandem multimers on the growth of *S. cerevisiae*; (**B**) statistical analysis of growth inhibition rate of *S. cerevisiae* after incubation for 48 h; (**C**) inhibitory effects of BmSPI39 tandem multimers on the growth of *C. albicans*; (**D**) statistical analysis of growth inhibition rate of *C. albicans* after incubation for 36 h. An equal volume of sterile 20 mmol/L PBS was used as a negative control and 100 mmol/L EDTA was applied as positive control. Error bars represent the standard error of the mean (*n* = 3). Different letters “a–e” indicate a significant difference between groups (*p* < 0.05), and one identical letter indicates no significant difference between groups (*p* < 0.05).

To further investigate the inhibitory effect of BmSPI39 tandem multimers on the growth of other fungi, the single-cell fungus *S. cerevisiae* and opportunistic human pathogen *C. albicans* were selected for fungal inhibition tests. The results showed that all forms of tandem proteins could significantly inhibit the growth of *S. cerevisiae* (Figure 6A,B) and *C. albicans* (Figure 6C,D). When incubated for 48 h, the inhibitory rate of His_6_-SPI39-monomer on the growth of Saccharomyces cerevisiae was only 5.81%, while the inhibitory rates of His_6_-SPI39-dimer, His_6_-SPI39-trimer, His_6_-SPI39-tetramer, His_6_-SPI39L-dimer, His_6_-SPI39L-trimer and His_6_-SPI39L-tetramer were 10.45%, 32.28%, 30.74%, 28.76%, 28.94% and 27.77%, respectively (Figure 6B). After incubation for 36 h, the inhibitory rates of His_6_-SPI39-monomer, His_6_-SPI39-dimer, His_6_-SPI39-trimer, His_6_-SPI39-tetramer, His_6_-SPI39L-dimer, His_6_-SPI39L-trimer and His_6_-SPI39L-tetramer against *C. albicans* were 16.97%, 15.28%, 10.35%, 11.43%, 11.97%, 20.10% and 15.40%, respectively (Figure 6D). His_6_-SPI39L-trimer had the strongest inhibitory effect on *C. albicans* among all the tandem multimeric proteins. These results indicate that BmSPI39 tandem multimers have certain inhibitory effects on *S. cerevisiae* and *C. albicans*, and the inhibitory ability of BmSPI39 against these two fungi can be enhanced by tandem multimerization.

## 4. Discussion

In this study, we successfully obtained the active tandem multimeric proteins of BmSPI39 by means of protein engineering and confirmed that tandem multimerization can not only greatly improve the structural homogeneity of BmSPI39 recombinant protein but can also improve the inhibitory activity of BmSPI39 protein against subtilisin and proteinase K. The tandem multimerization of BmSPI39 can also enhance its inhibitory ability against *B. bassiana*, single-cell fungus *S. cerevisiae* and opportunistic human pathogen *C. albicans*.

Protease inhibitors are the main regulators of protease catalytic activity in vivo, which can bind protease molecules and inhibit their physiological activities. Protease inhibitors play an important role in many physiological processes such as digestion, coagulation, phenoloxidase cascade, cell migration and inflammatory reaction [29,30,31,32,33]. The multimerization of proteins is a common phenomenon in living organisms. Many receptors, fungal immunomodulatory proteins, proteases and so on tend to exist and function in the form of multimers. Such a multimerization phenomenon has also been found in protease inhibitors, which are important for regulating the advanced structure and biological activity of protease inhibitors [34]. Ecotin is a serine protease inhibitor that can be produced by hundreds of microorganisms, including pathogens. Ecotin has a very broad inhibitory specificity for almost all serine proteases in the chymotrypsin, trypsin and elastase superfamily, which protects the microorganism from the host immune response [35,36,37]. It was found that the dimerized Ecotin can combine with protease to form heterotetramers with three distinct interfaces [35]. Cystatin C, a cysteine protease inhibitor, plays an important role in various physiological and pathological processes such as vascular remodeling and inflammation, and its activity can be regulated by changing the multimerization state [38,39]. DM43 is a homodimerized metalloproteinase inhibitor isolated from the serum of *Didelphis marsupialis*, which binds noncovalently to the metalloproteinase Jararhagin from *Bothrops jararaca* snake venom and effectively neutralizes its toxicity [40]. Studies have shown that dimerization is critical in determining the structure and stability of the DM43 protein, thus adapting its conformation to a diverse range of environments and binding proteins [41].

Our previous studies found that the TIL-type protease inhibitors BmSPI38 and BmSPI39 in silkworm could inhibit the invasion of pathogenic fungi by inhibiting its virulence protease [18,19]. The recombinant proteins encoded by either single-copy BmSPI38 or single-copy BmSPI39 genes have poor homogeneous in vitro and are prone to multimerization, forming dimers, trimers and tetramers [22,23]. Western blot results showed that the physiological forms of BmSPI38 and BmSPI39 in various tissues were mainly tetramer and a small amount of trimer, suggesting that multimerization is extremely important for their physiological functions [23]. In this study, the structural homogeneity and expression level of recombinant BmSPI39 protein was greatly improved using the strategy of tandem gene fusion expression (Figure 2 and Figure 3). The construction of tandem expression vectors can effectively improve the expression level and structural stability of small molecular recombinant proteins (peptides) and shield the harmful effect of toxic proteins on the host, so it is widely used [42,43,44,45,46,47,48,49]. Thymosin beta4 (Tβ4) is one of the major actin regulatory factors in the human body, which has a wide range of biological activities, and it is closely related to cytoskeletal balance, inflammatory response, angiogenesis, vascular regeneration, cell regulation and corneal and myocardial repair. 4×Tβ4 protein was successfully expressed in *E. coli* and demonstrated similar or better activity than the existing commercial Tβ4 protein [42]. Six copies of angiotensin I-converting enzyme inhibitory peptide (ACE-IP) genes were concatenated and inserted into an expression vector to achieve the fusion expression of the tandem peptide in *E. coli* BL21 (DE3) pLysS [44]. In addition, tandem expression also provided a convenient and economical method for the large-scale production of the antidiabetic drug *Momordica charantia* peptide MC6 [48].

Previous studies have confirmed that BmSPI39 can strongly inhibit subtilisin, proteinase K, *Aspergillus melleus* protease and the *B. bassiana*-sourced virulent protease CDEP-1 [19]. In this study, it was found that tandem multimerization could greatly improve the inhibitory activity of BmSPI39 against subtilisin. It should be noted that although His_6_-SPI39-monomer showed significantly weaker inhibitory activity against proteinase K than His_6_-SPI39-trimer, His_6_-SPI39L-dimer and His_6_-SPI39L-trimer, it was stronger than His_6_-SPI39-trimer, His_6_-SPI39-tetramer and His_6_-SPI39L-tetramer, suggesting that each tandem protein has a certain selectivity for the inhibition of different proteases. To date, there is no advanced structural data on BmSPI39 binding to proteases, and the formation mechanism of BmSPI39 multimers and the specific action mechanism on different proteases need to be further studied.

Previous studies found that BmSPI38 and BmSPI39 can not only inhibit the harmful melanization induced by *B. bassiana* virulence protease but also the conidial germination of *B. bassiana*, thereby improving the survival rate of the silkworm [18,19]. This study further confirmed the inhibition of BmSPI39 on the conidial germination of *B. bassiana*, and for the first time found that tandem multimerization could effectively enhance its inhibitory ability on the conidial germination of *B. bassiana* (Figure 5). We briefly summarized the reported multimerization studies of cysteine protease inhibitors, serine protease inhibitors and metalloprotease inhibitors and found that most of the protease inhibitors function as dimers and that the higher-order multimeric forms are further oligomerized based on the dimeric structure [34]. Dimerization is also present for many serine protease inhibitors with antifungal effects. It was found that the serine protease inhibitor BmSPI51 in the silkworm cocoon shell can significantly inhibit the spore growth of *B. bassiana*, *C. albicans* and *S. cerevisiae* [50]. The SDS-PAGE results showed that the apparent molecular weight of BmSPI51 recombinant protein obtained by the prokaryotic expression technique was about 12 kDa, which was consistent with its dimer size, suggesting that it may function as a dimer [50]. In addition, corn trypsin inhibitor (TI) with a molecular weight of about 14 kDa can inhibit the conidial germination and mycelial growth of plant pathogenic fungi. The SDS-PAGE results showed that the recombinant TI protein expressed in *E. coli* mainly existed in the form of a monomer, and a few existed in the form of a dimer [51]. Whether such dimerization will increase the activity and stability of a recombinant TI protein remains unclear, and the molecular mechanism of dimerization also requires further investigation.

As is well known, fungal infection has become one of the major threats to global public health, and problems such as toxic effects and drug resistance brought by existing antifungal drugs are increasingly prominent. Therefore, it is urgent to find new, efficient and safe antifungal drugs. *C. albicans* is the most common opportunistic pathogen among invasive fungi. In this study, we found that the BmSPI39 protein had an obvious inhibitory effect on *C. albicans*, and tandem multimization could significantly enhance its antifungal ability. Although many studies have confirmed the antifungal effect of *B. mori* protease inhibitors, their antifungal spectrum and specific antifungal mechanism are not fully understood, and more experimental data are still needed to support them. The development and application of protease inhibitors with antifungal effects need to be strengthened. It should be noted that His_6_-SPI39-monomer is much more effective in inhibiting *C. albicans* than *S. cerevisiae*, which may be related to its strong ability to inhibit proteinase K (Figure 4 and Figure 6). The types and abundance of proteases secreted by different fungi are very different during growth. Proteinase K used in this study is a powerful proteolytic enzyme derived from *C. albicans*, which can digest natural keratin and belongs to the serine protease of the Peptidase S8 family.

## 5. Conclusions

In this study, we successfully achieved the soluble expression of the tandem multimers of the silkworm protease inhibitor BmSPI39 in *E. coli* and confirmed that the tandem multimerization could significantly improve the structural homogeneity and antifungal ability of BmSPI39. This study will not only help to deepen people’s understanding of the action mechanism of BmSPI39 and provide an important theoretical basis and new strategies for cultivating antifungal transgenic silkworm materials, but it will also promote its exogenous production and development and application in the medical field.

## Figures and Tables

**Table 1:** Primers required for expression vector construction.

Primers	Sequence (5′ → 3′)
*BmSPI39*-*Nde* I-*Bam*H I-F	CGCCATATGGGCGGATCCTTTGAAAAAGATTGTCCTGAGAATTCT
*BmSPI39*-*Not* I-R	ATTTGCGGCCGCTTATGACTGTTGTTTATGGAAACAGTTG
*BmSPI39*-*Bgl* II-R	GAAGATCTTGACTGTTGTTTATGGAAACAGTTGAC
*BmSPI39-L*-*Bgl* II-R	GAAGATCTTGAGCCACCACCGCCTGAGCCACCACCGCCTGAGCC ACCACCGCCTGACTGTTGTTTATGGAAACAGTTGAC

*Nde* I (CATATG), *Bam*H I (GGATCC), *Not* I (GCGGCCGC) and *Bgl* II (AGATCT) restriction sites are underlined. The coding sequence of linker (L) is highlighted in gray.

## Data Availability

Not applicable.
